# Design of a Plant-Based Smoothie: Exploiting Ingredient Complementarity for a Diversified (Poly)phenolic Profile Quantified by Targeted LC-MS/MS Analysis

**DOI:** 10.3390/foods15081293

**Published:** 2026-04-09

**Authors:** Cristina Matías, Cristina Del Burgo-Gutiérrez, María-José Sáiz-Abajo, María-Paz De Peña, Iziar A. Ludwig, Concepción Cid

**Affiliations:** 1 Department of Nutrition, Centre for Nutrition Research, Food Science and Physiology, Faculty of Pharmacy and Nutrition, University of Navarra, C/Irunlarrea 1, 31008 Pamplona, Spain; cmatias@cnta.es (C.M.); cdel.7@unav.es (C.D.B.-G.); mpdepena@unav.es (M.-P.D.P.); ccid@unav.es (C.C.); 2 National Centre for Food Technology and Safety (CNTA), 31570 San Adrián, Spain; mjsaiz@cnta.es; 3 Instituto de Nutrición y Salud (INS), Universidad de Navarra, Campus Universitario, 31009 Pamplona, Spain; 4 Navarra Institute for Health Research (IdiSNA), 31008 Pamplona, Spain

**Keywords:** (poly)phenols, bioactive compounds, fruit and vegetable-based smoothies, functional beverages, LC-MS/MS

## Abstract

Smoothies represent a promising vehicle for increasing fruit and vegetable consumption and bioactive diversity. However, their formulation often lacks a rigorous analytical validation of phytochemical complementarity. This study establishes a methodological framework for the design of potential functional plant-based beverages, centered on a high-resolution LC-MS/MS-driven strategy. Through a targeted screening of 57 (poly)phenolic compounds, a precise phytochemical mapping of diverse botanical matrices was performed to optimize ingredient selection based on chemical diversity rather than empirical blending. A novel formulation combining Granny Smith apple, green celery, dried green chicory, and peppermint leaves was developed to maximize both bioactive density and structural variety. The resulting matrix achieved a total (poly)phenol concentration of 2947.68 ± 5.17 µg/g dm, encompasses six major subclasses: flavan-3-ols, hydroxycinnamic acids, flavanones, flavonols, flavones, and dihydrochalcones. The results demonstrate that analytical fingerprinting allows for the strategic enrichment of food systems, ensuring a highly characterized and diversified phenolic spectrum. This research shifts the focus toward the evidence-based molecular design of health-promoting foods with verified nutritional properties.

## 1. Introduction

Adherence to a plant-based diet has shown a preventive role against the onset of several pathologies, encompassing chronic and age-related diseases [1,2]. Although the World Health Organization (WHO) emphasizes the importance of fruits and vegetables consumption, the daily intake is lower than the minimum recommended (at least five portions or 400 g per day) to decrease the risk of developing different disorders [3]. The beneficial effect of a plant-based diet is, at least partly, attributed to their high content of phytochemicals, including (poly)phenols.

(Poly)phenols are secondary metabolites in plants that contain at least one aromatic ring with one or more hydroxyl groups. Based on their structure, (poly)phenols are divided into two main families: flavonoids and non-flavonoids. Flavonoids have a C6-C3-C6 structure, consisting of two aromatic rings linked together by a central oxygenated heterocyclic ring. Based on the oxidation grade of the heterocyclic ring, flavonoids are subdivided into flavonols, flavones, isoflavones, flavanones, anthocyanidins and flavan-3-ols. Non-flavonoids encompass phenolic acids, stilbenes and lignans. Among these, phenolic acids are the most abundant, comprising benzoic acids (C6-C1) and cinnamic acids (C6-C3). In addition, these naturally occurring phytochemicals can be found in free form, known as aglycones, or conjugated to sugar or acid moieties, referred to as glycosides and esters, respectively [4,5].

(Poly)phenols stand out for their antioxidant and anti-inflammatory activities. Therefore, these compounds have been extensively investigated as key constituents in the potential prevention of diseases closely related to oxidative stress and inflammation, such as neurodegenerative pathologies, cardiovascular disorders, cancer, and several metabolic-related alterations [6,7]. Given the wide range of (poly)phenol classes present in food/beverages, the specific (poly)phenols responsible for beneficial in vivo effects remain unknown.

Currently, there is a growing awareness among consumers of the relationship between lifestyle and overall health. As a result, the market has experienced rapid growth towards new functional foods, including beverages. These products not only contain health-promoting ingredients but also offer appealing organoleptic properties and convenient, ready-to-drink formats. Therefore, fruit- and vegetable-based smoothies could fulfill the demand of this health-conscious population while offering a practical way to increase fruit and vegetable consumption [8] and, consequently, dietary (poly)phenol intake.

However, a significant challenge in this field arises from the fact that the (poly)phenol content of food matrices, including plant-based smoothies, is often assumed without prior analytical verification, or only the total (poly)phenol content is evaluated [9,10,11], rather than specific profiles. The widespread use of the Folin–Ciocalteu assay to estimate total phenolic content is inherently non-specific, as the reagent reacts with various non-phenolic reducing agents present in the vegetable matrix—such as Vitamin C, sugars, and organic acids—thereby leading to a systematic overestimation of the actual phenolic levels. Furthermore, some studies [8,12,13] with HPLC rely on tentative identifications based on limited reference standards, expressing concentrations as equivalents of a few (poly)phenolic compounds—a practice that inherently compromises quantitative accuracy by potentially overestimating or underestimating the actual (poly)phenol levels and represents a major limitation in characterizing the specific content of each individual compound present in the matrix. Adding to this challenge, different (poly)phenol families or specific compounds may exert distinct roles in mediating health benefits, potentially leading to varying degrees of positive and synergistic effects on the gut microbiota. In fact, a recent study suggests that consuming a higher quantity and wider diversity of flavonoids is better for longer-term health than either component alone [14]. Furthermore, many dietary intervention studies suffer from a lack of comprehensive food analysis, particularly regarding specific (poly)phenol content, with most research predominantly focusing on a limited set of individual flavonoids [4].

Therefore, this research aimed to establish a robust methodological framework for the design of potential functional plant-based matrices, using a novel fruit and vegetable-based smoothie as a model. The core originality of this study lies in the systematic integration of high-resolution LC-MS/MS profiling to guide and validate the assembly of a highly diversified (poly)phenolic spectrum (including both flavonoids and non-flavonoids). Rather than a simple ingredient blend, the study focused on the analytical mapping of chemical complementarity between matrices, providing a precise molecular fingerprint of the resulting bioactive content while ensuring sensory acceptance of the smoothie. This approach establishes a reproducible scientific benchmark for fortifying food systems through precise phytochemical characterization, moving beyond empirical formulation toward evidence-based work.

## 2. Materials and Methods

### 2.1. Chemical and Reagents

Pure standards of the following (poly)phenolic compounds were supplied by Sigma-Aldrich (Darmstadt, Germany): 2,5-dihydroxybenzoic acid, 3,4-dihydroxybenzoic acid, 3,4,5-trihydroxybenzoic acid (gallic acid), 3,5-dimethoxy-4-hydroxybenzoic acid (syringic acid), 4′-hydroxycinnamic acid (*p*-coumaric acid), 3′,4′-dihydroxycinnamic acid (caffeic acid), 4′-hydroxy-3′-methoxycinnamic acid (ferulic acid), 5-*O*-caffeoylquinic acid, 4-*O*-caffeoylquinic acid, 3,5-*O*-dicaffeoylquinic acid, 3,4-*O*-dicaffeoylquinic acid, 4,5-*O*-dicaffeoylquinic acid, 2-(3′,4′-dihydroxyphenyl)ethanol (hydroxytyrosol), (−)-epicatechin, (+)-catechin, kaempferol, kaempferol-3-*O*-rutinoside, quercetin, quercetin-3-*O*-rhamnoside, quercetin-3-*O*-glucoside, quercetin-3-*O*-rutinoside, isorhamnetin, isorhamnetin-3-*O*-rutinoside, apigenin, apigenin-7-*O*-glucoside, apigenin-7-*O*-glucuronide, apigenin-7-*O*-rutinoside, apigenin-8-C-glucoside, apigenin-6,8-C-diglucoside, luteolin, luteolin-7-*O*-glucoside, luteolin-8-C-glucoside, luteolin-7-*O*-glucuronide, diosmetin, diosmetin-7-*O*-glucoside, naringenin-7-*O*-rutinoside and isosakuranetin-7-*O*- rutinoside.

Procyanidin dimer B2, procyanidin dimer B1, quercetin-3-*O*-arabinoside, kaempferol-7-*O*-glucoside, isorhamnetin-3-*O*-glucoside, phloretin-2′-*O*-glucoside, eriodictyol and hesperetin-7-*O*-rutinoside were purchased from Extrasynthese (Genay, France).

2-*O*-Caffeoyl-3-(3,4-dihydroxyphenyl)lactic acid (rosmarinic acid), 2,3-*O*-dicaffeoyltartaric acid (chicoric acid), epigallocatechin, procyanidin trimer C1, kaempferol-3-*O*-glucuronide, quercetin-3-*O*-xyloside, quercetin-3-*O*-galactoside, phloretin, apigenin-7-(2-*O*-apiosylglucoside), diosmetin-7-*O*-rutinoside, eriodictyol-7-*O*-rutinoside and hesperetin were obtained from MedChemExpress (Sollentuna, Sweden).

Acetonitrile and formic acid (both LC-MS grade) were supplied by Scharlau (Barcelona, Spain). Methanol (LC-MS grade) was bought from PanReac AppliChem (Darmstadt, Germany).

### 2.2. Sample Preparation

Apples (*Malus domestica* cv. Granny Smith), green celery (*Apium graveolens*), green chicory (*Cichorium intybus* L.), borage (*Borago officinalis*), Swiss chard (*Beta vulgaris* L. var. cicla) and lemon (*Citrus limon*) were purchased from a local market in Calahorra, Spain. Dried peppermint leaves (*Mentha piperita*) were bought from the company Origeens (Estrées-Saint Denise, France).

Fresh ingredients were washed and cut into small pieces prior to smoothie preparation. Both edible parts of Granny Smith apple, flesh and peel, were used. Apple and lemon juices were freshly squeezed. Green chicory was lyophilized in a Cryodos-80 freeze dryer (Telstar, Terrasa, Spain). Dried chicory and peppermint were added to the formulation as fine powder. Each smoothie formulation (500 g) was prepared by blending the ingredients at maximum speed for 1 min using a blender (Power Black Titanium 2000 Pro, Cecotec, Valencia, Spain). Lemon juice was added until the pH reached 3.4. All ingredients and smoothies were freeze-dried and stored at −18 °C until further (poly)phenol analysis by LC-MS/MS.

### 2.3. Extraction of (Poly)phenolic Compounds

(Poly)phenols were extracted from ingredients and smoothies following the method of Domínguez-Fernández et al. [15], with minor modifications. The extraction made use two different methanol/water ratios to maximize the extraction yield across the structural variety of the target analytes. Briefly, 25 mg of freeze-dried samples was mixed with 0.5 mL of methanol/acidified Milli-Q water (0.1% formic acid) (80:20 *v*/*v*). Then, samples were vortexed, sonicated for 15 min and centrifuged at 18,626× *g* for 10 min (Mikro 200, Hettich, Tuttlingen, Germany). The supernatant was collected and the residue was subjected to a second extraction by adding 0.25 mL of methanol/acidified Milli-Q water (0.1% formic acid) (50:50 *v*/*v*). Afterwards, they were vortexed, sonicated for 15 min and centrifuged at 18,626× *g* for 10 min. The supernatant was pooled with the previous one and filtered through a 0.22 µm PVDF syringe filter prior to LC-MS/MS analysis. Samples were extracted, properly diluted and analyzed in triplicate.

### 2.4. Identification and Quantification of (Poly)phenolic Compounds by LC-MS/MS

The (poly)phenol analysis followed the previously described method by Domínguez-Fernández et al. [15], with some modifications. The instrumentation consisted of an HPLC unit model 1200 (Agilent Technologies, Palo Alto, CA, USA) coupled to a triple quadrupole linear ion trap mass spectrometer (3200 Q-TRAP, AB SCIEX, Madrid, Spain).

Chromatographic separation was carried out using a reverse-phase CORTECS C18 column (3 mm × 75 mm, 2.7 µm) from Waters (Barcelona, Spain). The binary mobile phase consisted of acidified Milli-Q water (0.1% formic acid) (solvent A) and acetonitrile (solvent B). The flow rate was set at 0.6 mL/min, the injection volume was 5 µL and the column oven temperature was kept at 30 °C. Elution was performed following the gradient as described by Domínguez-Fernández et al. [15].

After chromatographic separation, a triple quadrupole mass spectrometer equipped with a heated electrospray ionization source was employed for identification and quantification. Spectral data were acquired in negative ionization mode based on previous work by our group [15,16]. The turbo heater was kept at 600 °C and the ion spray voltage was set at −3500 V. Nitrogen was used as a nebulizing, turbo heater and curtain gas, and it was set at the pressure of −60, −65 and −35 psi, respectively. A preliminary analysis was conducted to identify the (poly)phenolic compounds characteristic of each sample. Full scan mode analysis was carried out in the mass range (*m*/*z*) from 100 to 1000. Subsequently, a selective product ion analysis (MS/MS) was conducted, to determine the fragmentation pattern of each molecular ion. Ion multiple reaction monitoring (MRM) mode analysis was selected for (poly)phenol quantification in the ingredients and the smoothie.

MS conditions were optimized by direct infusion of pure standard solutions (1 µg/mL) into the ion source at a constant flow rate of 10 µL/min using a syringe pump (1001 TLL SYR, Hamilton, Giarmata, Romania). The MS parameters automatically optimized for each analyte were MRM transition (transition from precursor ion to product ion) and Collision Energy (CE). Identification was performed by comparing the retention time, the molecular ion and the MS/MS spectrum with those of pure standards. Details of retention time, MRM ion transitions and Collision Energy (CE), as well as chromatograms, are reported in the Supplementary Data (Table S1, Figures S1–S8). Quantification was achieved by external calibration curves of pure standards. All calibration curves showed determination coefficients (R^2^) > 0.99.

Data acquisition and processing were performed using Analyst software 1.6.3 (AB SCIEX, Framingham, MA, USA). Results were expressed in micrograms of each (poly)phenolic compound per gram of sample dry matter (µg/g dm). The standardized nomenclature for (poly)phenols proposed by Kay et al. [17] was used in the present research.

### 2.5. Sensory Evaluation of Smoothies

A group of ten panelists performed a descriptive analysis according to the sensory sheet detailed in the Supplementary Materials Table S2. Panelists were selected and trained according to ISO 8586 [18]. Smoothies were presented individually to each panelist in a randomized order. The sensory evaluation was conducted in a standardized environment following the guidelines of ISO 8589 [19]. Smoothies were assessed based on appearance (color intensity and absence of defects), odor (intensity and off-odor), taste/flavor (intensity, sweetness, acidity, bitterness, persistence in mouth, astringency, and off-taste/off-flavor) and texture (consistency and presence of lumps, fibers, sandiness, etc.). These attributes were scored using a numerical scale from one to seven (1, absence; 2, light; 3, moderate; 4, adequate presence; 5, intense; 6, very intense; 7, extremely intense), except for color intensity, which was scored based on a specific scale as follows: 1, yellow; 2, yellow-greenish; 3, green; 4, dark green; 5, brownish-green; 6, brown. Hedonic analysis of appearance, odor, taste/flavor, texture and global acceptance of selected smoothies was evaluated by the same panelists with a nine-point hedonic scale ranging from extremely disliked (1) to extremely liked (9). Scores less than 5 imply rejection of the samples.

### 2.6. Color Parameters

The chromatic parameters were measured with a Chroma Meter CM-5 (Konica Minolta, Tokyo, Japan) according to the CIE L* a* b* scale. The instrument was calibrated with white and black color standards prior to the analysis. The chromameter was set up for 10^0^ observer angle and D65 illuminant. The chromatic parameters of L*, a*, and b* represent lightness (0 = black, 100 = white), red/green value (−a* = greenness, +a* = redness) and yellow/blue value (−b* = blueness, +b* = yellowness), respectively. These parameters were employed to obtain the Chroma (C = [a*^2^ + b*^2^]^1/2^) and Hue degree (h^0^ = arctangent [b*/a*]), which represent color saturation or intensity and tonality, correspondingly.

## 3. Results and Discussion

Smoothies are blended beverages composed of more than one fruit and/or vegetable. These ready-to-drink products are an excellent way to increase not only the intake of fruit and vegetables, but also the diversity of them. In this regard, selecting specific yet varied plant-based matrices may result in smoothies with a broader range of naturally present (poly)phenols (flavonoids and non-flavonoids), thus enhancing their potential health benefits. Therefore, in this study, special attention was first paid to the content and the variety of the phenolic profile of the ingredients selected for the smoothie design. Then, a LC-MS/MS method was developed to cover the specific (poly)phenols present in the selected plant matrices, based on a preliminary full scan analysis and literature review [20,21,22,23,24,25,26] and included 57 (poly)phenolic compounds. Pure standards were used for identification and quantification of each individual (poly)phenolic compound, a necessary requirement for their correct assessment by mass spectrometric methods [27]. Beyond their health-promoting ingredients, smoothies are valued for their appealing sensory attributes, such as a fresh appearance, pleasant aroma, and smooth texture. Consequently, the formulation of the (poly)phenol-rich matrices was optimized based on sensory evaluation to ensure a balanced and acceptable plant-based ready-to-drink product.

Formulations of green smoothies usually contain a liquid base composed of fruits and vegetables enriched with leafy vegetables and herbs. Apple, one of the most consumed fruits worldwide, is well-known for fruit-based formulations and a popular main ingredient in most commercialized smoothies on the Spanish market [12]. Additionally, the adequacy of apple juice for the development of innovative smoothies [8] and beverages [13], together with other fruits and vegetables, has been previously reported. Thus, in the present study, apple was selected as a key fruit for the development of apple-based green smoothies. Among green apple varieties, the Granny Smith apple was chosen because of its lower pH, which makes it less susceptible to browning during blending, compared to other apple varieties [28].

Due to its high water content, green celery is usually added to green smoothies, yet as a minor constituent compared to apple [12], or it is consumed as juice [29]. This vegetable has been cultivated since ancient times and has gained much attention among nutritionists and vegan consumers in recent years due to its promising phytochemical composition, which includes some simple phenolic acids, and flavonoids not present in apples [30].

Green smoothies may also be enriched with leafy vegetables including flavoring agents (such as herbs), which might improve the (poly)phenolic diversity because of their abundance in phenolic compounds [31], as well as providing aromatic flavors able to mask the astringency of other vegetables, like green celery, used in the formulations. Based on a literature review, green chicory [21,25], borage [23], Swiss chard [20,24] and peppermint [22,26] were selected as leafy green vegetables due to them encompassing diverse (poly)phenol profiles. Most of these studies were limited to some individual (poly)phenolic compounds but did not include a comprehensive characterization.

### 3.1. (Poly)phenolic Profile of Green Liquid Base Ingredients—Apple and Green Celery

**Figure 1:**
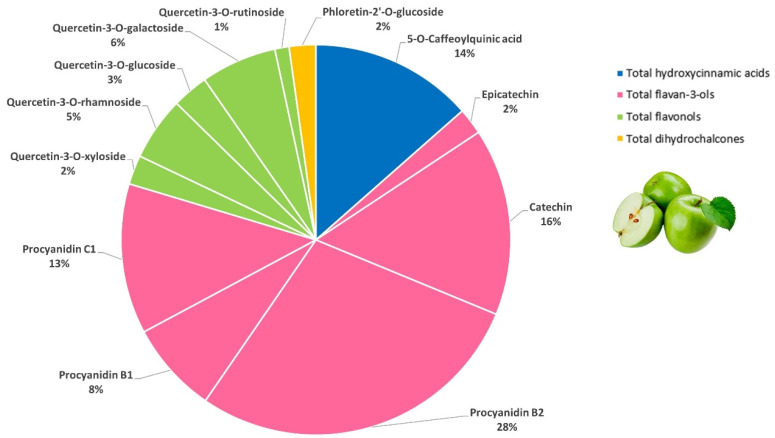
Composition of the main (poly)phenolic compounds quantified in Granny Smith apple (>0.5% of the total (poly)phenols).

A total of fifteen (poly)phenols were identified and quantified in Granny Smith apple by LC-MS/MS (Table 1). Flavonoids were the main (poly)phenolic compounds in Granny Smith apple, accounting for 86% of the total (poly)phenols quantified. The most representative compounds were flavan-3-ols, which comprised 67% of total phenolic compounds. Procyanidin B2 showed the highest amount, followed by catechin, procyanidin C1, procyanidin B1 and epicatechin. The second largest subclass was flavonols (17% of total phenolic compounds), with quercetin derivatives as dominant constituents. The highest level was detected in quercetin-3-*O*-galactoside, followed by quercetin-3-*O*-rhamnoside, quercetin-3-*O*-glucoside, quercetin-3-*O*-xyloside, quercetin-3-*O*-rutinoside and quercetin-3-*O*-arabinoside. Hydroxycinnamic acids were the third major group (14%) in Granny Smith apple, and their contribution was almost entirely due to 5-*O*-caffeoylquinic acid. The least abundant subclass identified was dihydrochalcones, which corresponded to 2% of total (poly)phenol content, with phloretin-2′-*O*-glucoside being the only representative detected. The importance of these derivatives lies in their almost exclusive occurrence in apples. Only a few other plant species are able to synthesize phloretin-2′-*O*-glucoside and, unlike *Malus domestica*, accumulate only very low amounts of this compound [32]. In summary, Granny Smith apple showed an assorted (poly)phenolic profile consisting of flavan-3-ols in monomeric, dimeric and trimeric forms, flavonols, hydroxycinnamic acids and dihydrochalcones (Figure 1). This characterization was in accordance with previous studies on apple (poly)phenolic contents [33,34].

**Figure 2:**
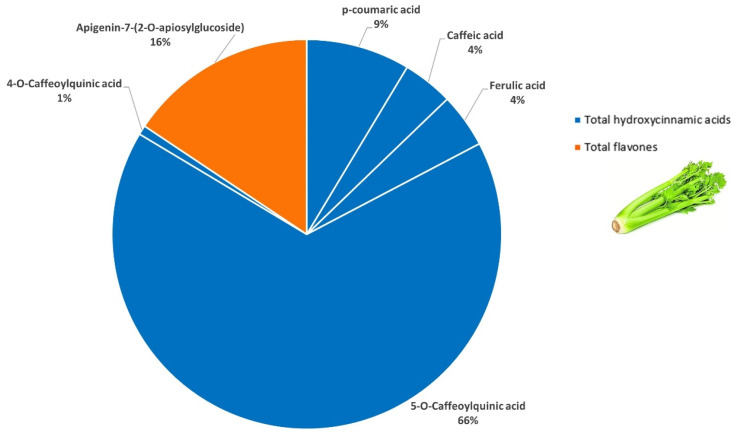
Composition of the main (poly)phenolic compounds quantified in green celery (>0.5% of the total (poly)phenols).

In green celery, a total of eight (poly)phenolic compounds were detected and quantified by LC-MS/MS (Table 1). Hydroxycinnamic acids were the most abundant (84%) (Figure 2) with 5-*O*-caffeoylquinic acid accounting for 66% of the total content, followed by minor quantities of p-coumaric, ferulic, caffeic and 4-*O*-caffeoylquinic acids. Apigenin-7-(2-*O*-apiosylglucoside) (aka apiin) stood out among flavones and represented the remaining 16% of total (poly)phenols. This characteristic flavonoid in celery has shown strong in vitro and in vivo antioxidant properties [35]. Overall, these results are in good agreement with previously published profiles [36,37] in which apigenin glycosides and hydroxycinnamic acids (caffeoylquinic, p-coumaric, ferulic and caffeic acids) were identified as the characteristic phenolic compounds in celery. Some flavonoids aglycones, such as apigenin, luteolin, and kaempferol, commonly observed in celery [30], were not detected in our study.

### 3.2. (Poly)phenolic Profile of Leafy Green Vegetables—Chicory, Peppermint, Borage, and Swiss Chard

**Figure 3:**
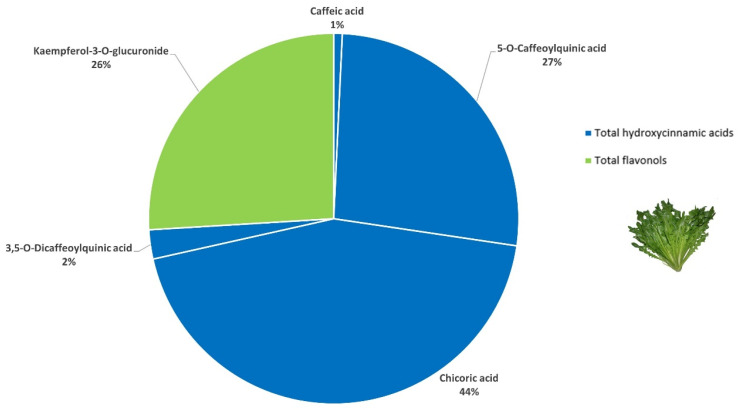
Composition of the main (poly)phenolic compounds quantified in green chicory (>0.5% of the total (poly)phenols).

A preliminary LC-MS/MS screening of four leafy vegetables identified green chicory (6805.08 µg/g dm) and peppermint (66,551.02 µg/g dm) as the most promising matrices. Conversely, borage (41.02 µg/g dm) and Swiss chard (57.33 µg/g dm) were discarded from the green smoothie formulation because their total phenolic content was >100-fold lower compared with the other samples and because their (poly)phenolic profile did not contribute any distinctive (poly)phenol subfamily (Table S3). A total of ten (poly)phenolic compounds were found in green chicory by LC-MS/MS (Table 1). Hydroxycinnamic acids were the most abundant (poly)phenols (74%), in particular, 2,3-*O*-dicaffeoyltartaric acid (chicoric acid) and 5-*O*-caffeoylquinic acid. Flavonols were the second largest subclass in green chicory (26%) and almost entirely attributed to kaempferol-3-*O*-glucuronide. These three (poly)phenols were found in high concentrations and accounted for 97% of the total (poly)phenolic compounds quantified (Figure 3).

In general, these results are in good agreement with those previously reported [25,38], in which hydroxycinnamic acids and kaempferol derivatives were key constituents in chicory varieties. However, luteolin-7-*O*-glucuronide and apigenin-7-*O*-glucuronide were not detected in green chicory in the present study. This finding contrast with that of Heimler et al. [38], who observed significant concentrations of flavone derivatives in the green variety “Spadona” cultivated under different growing conditions, in particular, glucuronide derivatives of luteolin (from 0.98 to 3.33 mg/g fresh weight) and apigenin (from 0.18 to 0.68 mg/g fresh weight). These discrepancies are likely attributable to differences in the quantification methods applied. While the present study utilized LC-MS/MS with pure reference standards, Heimler et al. [38] employed LC-DAD and quantified flavones using structurally similar equivalent compounds, which may lead to inaccurate quantification.

The (poly)phenolic profile of peppermint comprised a total of thirty-six compounds according to LC-MS/MS analysis (Table 1). Peppermint’s major (poly)phenols were flavanones (75% of total (poly)phenols), mainly those derivatives with the disaccharide rutinose attached at position 7. By far, the most representative was eriodictyiol-7-*O*-rutinoside, which was found in a noteworthy amount (43,473.70 ± 5436.81 µg/g dm). Other flavanones present in remarkable quantities were hesperetin-7-*O*-rutinoside, eriodictyol and naringenin-7-*O*-rutinoside. These findings are consistent with previous studies [39], where eriodictyol-7-*O*-rutinoside was identified as the major compound in peppermint-based preparations.

The second most abundant group in peppermint was hydroxycinnamic acids (12% of total (poly)phenols), predominantly due to the presence of 2-*O*-caffeoyl-3-(3′,4′-dihydroxyphenyl)lactic acid (aka rosmarinic acid). This is in line with other studies [26], in which rosmarinic acid was described as a characteristic antioxidant in peppermint. Moreover, dihydroxybenzoic and dicaffeoylquinic acids were detected in peppermint in the present study to an extent that was comparable with previous findings on peppermint (poly)phenolics [40,41]. However, as far as we know, 4,5-*O*-dicaffeoylquinic, 3,5-*O*-dicaffeoylquinic, and 2,5-*O*-dihydroxybenzoic acids were detected in peppermint for the first time.

Flavones in peppermint accounted for 12% of total (poly)phenols, with luteolin-7-*O*-glucuronide, diosmetin-7-*O*-rutinoside and apigenin-7-*O*-rutinoside quantified as the most abundant. Furthermore, diosmetin-7-*O*-glucoside, present in low quantities, was also identified for the first time in peppermint leaves. The least abundant group characterized comprised flavonols almost only due to the contribution of isorhamnetin-3-*O*-rutinoside, which, together with kaempferol-7-*O*-glucoside, which was present in minor quantities, were reported for the first time in the present study in peppermint.

**Figure 4:**
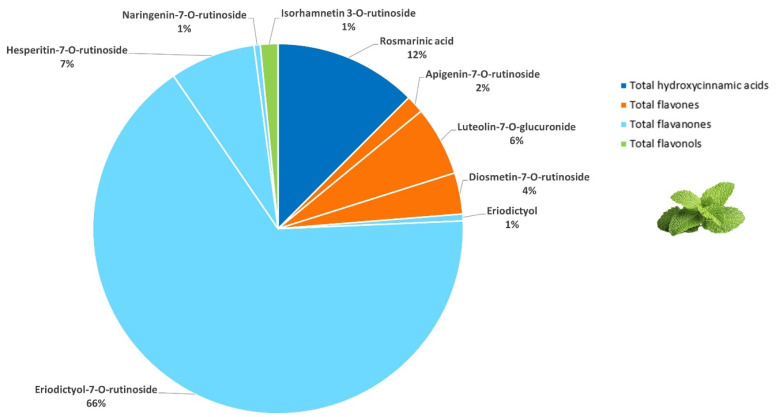
Composition of the main (poly)phenolic compounds quantified in peppermint (>0.5% of the total (poly)phenols).

Briefly, a highly varied (poly)phenolic profile was found in peppermint, mainly involving flavanones, hydroxycinnamic acids, flavones and flavonols (Figure 4). Among them, eriodictyol-7-*O*-rutinoside, rosmarinic acid and hesperetin-7-*O*-rutinoside were found in remarkable concentrations. Moreover, other derivatives linked to disaccharide rutinose or glucuronic acid were quantified in significant amounts, such as luteolin-7-*O*-glucuronide, diosmetin-7-*O*-rutinoside, apigenin-7-*O*-rutinoside and isorhamnetin-3-*O*-rutinoside. To our knowledge, seven (poly)phenolic compounds identified in the present study are herein reported for the first time in peppermint. Among these, isorhamnetin-3-*O*-rutinoside was found in a notable quantity (Table 1).

Based on this LC-MS/MS targeted approach, apple, green celery, green chicory and peppermint were selected for the design of (poly)phenol-rich smoothies. This selection was due to the high content and characteristic (poly)phenolic profile of each ingredient, which provided a diverse array of distinct compound families.

### 3.3. Green Smoothie Formulation and Selection

Several formulations combining apple, green celery, green chicory and peppermint in different proportions were prepared to obtain a green smoothie with a high and diverse content of (poly)phenolic compounds but also with attractive organoleptic properties. For this purpose, a descriptive sensory analysis was conducted to achieve the combination of these ingredients that led to the following desired sensory attributes: intense green color and homogeneous appearance with no phase separation, a fruit-like and fresh odor, a balanced sweet, bitter, and sour taste, and a ready-to-drink texture without undesirable fibers.

First, the ratio of apple and celery (liquid base ingredients) was adjusted to obtain a ready-to-drink product balanced in sweetness and astringency. Preliminary attempts combining whole apple and green celery resulted in excessively thick formulations. Therefore, apple was partially added as apple juice to achieve drinkable formulations without diluting the (poly)phenolic composition. As a result, ratios of apple from 30 to 42 g/100 g, apple juice from 30 to 37 g/100 g and green celery from 25 to 32 g/100 g led to a product with a drinkable and smooth consistency.

Afterwards, the ratio of leafy ingredients (chicory and peppermint) was optimized in order to maximize their content without compromising organoleptic attributes. These ingredients were added in dried form to prevent enzymatic browning and, consequently, (poly)phenol degradation. Green chicory showed a characteristic (poly)phenol profile with high contents of two exclusive derivatives—chicoric acid and kaempferol-3-*O*-glucuronide—among the plant matrices. Given its unique composition, trials were performed to balance maximum chicory incorporation with desirable sensory attributes. In the descriptive sensory evaluation, concentrations exceeding 1 g/100 g resulted in astringency scores above 4 (indicating a distinct presence). Therefore, a range of 0.5 to 1 g/100 g was selected to maximize chicory inclusion without compromising the flavor profile. Considering chicory’s elevated water content (94%), these dry matter values correspond to 8.3–16.7 g of fresh matter per 100 g of smoothie. In the case of dried peppermint, increasing amounts were tested, and the incorporation of 0.1 g/100 g of dried peppermint provided a more balanced flavor, without overpowering the other ingredients. Peppermint showed outstanding concentrations of flavanones (eriodictyol-7-*O*-rutinoside and hesperetin-7-*O*-rutinoside), as well as some hydroxycinnamic acids (rosmarinic acid) and flavones (luteolin-7-*O*-glucuronide, diosmetin-7-*O*-rutinoside and apigenin-7-*O*-rutinoside) not present in the other plant-based matrices used for smoothie formulations (Table 1). To ensure the contribution of these compounds while maintaining the desired sensory attributes, 0.1 g of dried peppermint per 100 g of smoothie was incorporated, corresponding to 1.7 g of fresh peppermint. Moreover, as blending of the ingredients ruptures plant cells and structures, the released (poly)phenols become highly susceptible to enzymatic oxidation. This process leads to browning and the subsequent degradation of phenolic compounds and loss of green color. To mitigate this, lemon juice was added as an acidulant to inhibit enzymatic browning [28], adjusting the formulation until a pH of 3.4 was achieved. As a result, five smoothies were formulated based on Granny Smith apple, green celery, green chicory and peppermint according to Table 2.

**Figure 5:**
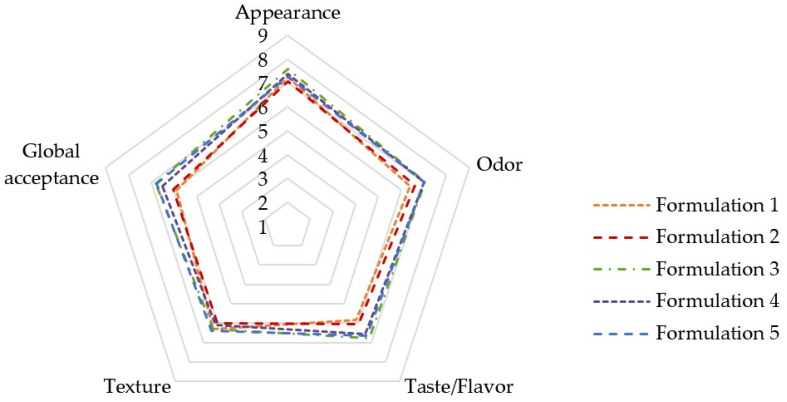
Sensory scores of the five smoothie formulations based on hedonic analysis.

To select the green smoothie formulation with the best sensory attributes, a hedonic analysis was performed (Figure 5). All smoothies were rated highly by the panel, with scores ranging from Like Slightly (6.6) to Like Very Much (7.6). Among them, formulation 3 obtained the highest scores in appearance (8.4), odor (7.8) and taste/flavor (7.6). Additionally, this formulation displayed good scores on texture (7.0) and global acceptance (7.6). Hence, this smoothie was selected for further (poly)phenolic characterization by LC-MS/MS. Color measurement of the selected formulation, performed according to the CIE L*a*b* scale, indicated a green color, showing the following chromatic values: Chroma, 14.17; Hue angle, 97.48°; L*, 39.90; a*, −1.84; and b*, 14.05.

### 3.4. (Poly)phenolic Profile of the Selected Green Smoothie Formulation

**Figure 6:**
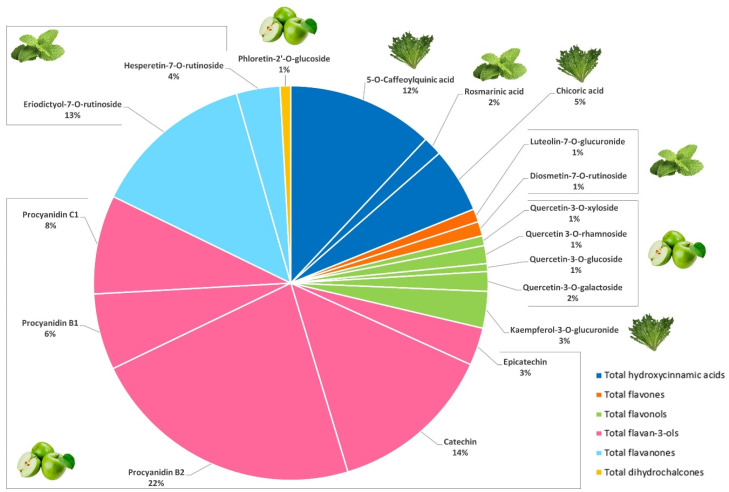
Composition of the main (poly)phenolic compounds quantified in the smoothie (>0.5% of the total (poly)phenols) and its main plant source.

A total of forty-three (poly)phenolic compounds were identified and accurately quantified in the smoothie using LC-MS/MS (Table 1). The phenolic profile of the selected green smoothie formulation was characterized by the most representative (poly)phenols of individual ingredients, involving six different subclasses: flavan-3-ols, hydroxycinnamic acids, flavanones, flavonols, flavones and dihydrochalcones (Figure 6).

Flavan-3-ols were the most abundant group, comprising 53% of the total (poly)phenols quantified in the smoothie, and were provided uniquely by the apple. Flavan-3-ols played an important role in the developed smoothie due to its high apple content (66.7 g/100 g considering both juice and whole fruit). Wu et al. [42] assessed the in vitro effect of Granny Smith apple procyanidin-rich extract (mainly B2) on inflammatory gastrointestinal disease, showing its beneficial role in preventing this disorder. Slightly higher concentrations of monomeric flavan-3-ols were found in the smoothie (387.00 ± 17.05 µg catechin/g dm and 88.52 ± 4.62 µg epicatechin/g dm) than in the apple itself (366.95 ± 7.81 µg catechin/g dm and 52.03 ± 2.46 µg epicatechin/g dm). The blending of ingredients to make the smoothie may favor the release of these compounds from the cell matrix as well as the breakdown of oligomeric proanthocyanidins structures, releasing monomeric units [12,33].

The second largest group in the smoothie was hydroxycinnamic acids (19% of total (poly)phenols). Among them, 5-*O*-caffeoylquinic, chicoric and rosmarinic acids were the most representative compounds. It is worth noting that, although all the selected ingredients contained 5-*O*-caffeoylquinic acid, its occurrence might be mainly attributed to chicory given its high concentration of this hydroxycinnamic acid (1807.62 ± 49.15 µg/g dm). Chicoric and rosmarinic acids were exclusively provided by chicory and peppermint, respectively. Several in vitro and in vivo studies suggested these hydroxycinnamic acids as potential antioxidants in the prevention of diet-related metabolic and neurodegenerative disorders [43,44,45,46].

Flavanones were the third most abundant group in the smoothie, representing 17% of total phenolic compounds, and were derived from peppermint. Eriodictyol-7-*O*-rutinoside and hesperitin-7-*O*-rutinoside were the main representative compounds in the smoothie. Despite the minimal inclusion of peppermint (0.1 g/100 g of dried leaves) in the formulation, these two flavones remained key constituents of the final (poly)phenolic profile. This is explained by the exceptionally high native concentrations of these compounds in peppermint, which allow them to exert a substantial influence on the smoothie’s composition even at low inclusion levels (Table 1). Both flavanones have shown potential protective effects on oxidative stress and inflammation and related diseases such as obesity and related metabolic disorders, cardiovascular disease, cancer and neurodegenerative disorders [47,48].

Flavonols represented 8% of total phenolic compounds in the smoothie. Among them, several quercetin glycosides and kaempferol-3-*O*-glucuronide were the predominant constituents. Regarding quercetin derivatives (which accounted for 5% of total (poly)phenols), quercetin-3-*O*-galactoside, quercetin-3-*O*-rhamnoside, quercetin-3-*O*-xyloside and quercetin-3-*O*-glucoside were derived exclusively from apple, while quercetin-3-*O*-glucosid was also present in peppermint. Kaempferol-3-*O*-glucuronide came exclusively from chicory and provided 3% of the total (poly)phenols quantified in the smoothie. Quercetin-3-*O*-galactoside and quercetin-3-*O*-rhamnoside have shown in vitro protective effects against inflammation and skin aging [49], whereas glucuronide derivatives of kaempferol have been proposed to improve glucose metabolism [50]. It should be noted that, although very low quantities of quercetin (1.19 ± 0.10 µg/g dm) and kaempferol (0.51 ± 0.06 µg/g dm) were quantified in the smoothie, these amounts were higher than those found in the ingredients themselves, peppermint (0.30 ± 0.03 µg quercetin/g dm) and chicory (0.38 ± 0.02 µg kaempferol/g dm), respectively. Similarly to monomeric flavan-3-ols units, this is likely due to the blending of the ingredients, which may facilitate the release of aglycones from the cell matrix through the breakdown of glycosylated and glucuronide derivatives [13].

Flavones constituted 2% of total (poly)phenolic compounds and were exclusively derived from peppermint. The most abundant derivatives were luteolin-7-*O*-glucuronide and diosmetin-7-*O*-rutinoside. Peppermint tea extracts, particularly flavone derivatives, have been associated with beneficial health effects on acute respiratory tract infections in in vitro studies [39].

The least abundant group in the smoothie was dihydrochalcones, represented solely by phloretin-2′-*O*-glucoside, which originated exclusively from apple. This (poly)phenolic compound (aka phloridzin) has been suggested as a natural potential candidate for treatment for type 2 diabetes and related metabolic disorders [51,52].

The total (poly)phenolic compounds quantified in the smoothie were 2947.68 ± 5.17 µg/g dm. The incorporation of dried green chicory and peppermint into an apple-based product was an effective strategy for enhancing (poly)phenol concentrations, leading to a 24% increase in the (poly)phenol content in relation to apple fruit (2381.25 ± 10.34 µg/g dm). Additionally, it allowed the enrichment of specific (poly)phenols, including flavanones, flavones, caffeoyltartaric and lactic acid derivatives (chicoric and rosmarinic acids, respectively), as well as kaempferol derivatives. Our findings are in line with Biegańska-Marecik et al. [13], who stated that the addition of dried products is a feasible strategy to enrich the (poly)phenol content. Moreover, dried (poly)phenol-rich ingredients may provide a convenient means of incorporating extracts into formulations in powdered form [31].

Limited literature is available on the in-depth LC-MS-based characterization of polyphenols in food ingredients and beverage formulations. However, our research aligns with the findings of Chou et al. [40], who characterized the (poly)phenolic profile in an herbal infusion and its ingredients (ginger, lemon, and mint) to promote the consumption of polyphenol-rich beverages. Although they identified seventy-three (poly)phenolic compounds by LC-MS, quantitative data was only available for ten. Notably, and consistent with results of the current work, mint exhibited the highest (poly)phenol content (113.14 mg/g) and variety (49 compounds). These findings further reinforce that peppermint is a promising ingredient for enhancing the polyphenol content of beverages, including smoothies. Furthermore, the green fruit and vegetable smoothie developed in the present study demonstrates a considerably higher (poly)phenol content (32.42 mg/100 g fresh smoothie) compared to other commercial smoothies (strawberry/banana and mango/passion fruit) previously characterized by in-depth LC-MS methods (7.69 and 9.36 mg/100 mL, respectively) [53]. Several authors have reported a total phenolic content, measured by Folin–Ciocalteu assay, from 39.1 to 150 mg gallic acid equivalent/100 g in different fruit smoothies [9,10,11,54]. Unfortunately, as previously mentioned, a direct comparison of our results—obtained through comprehensive LC-MS profiling—with those from studies using either the Folin–Ciocalteu assay or tentative identifications quantified as equivalents [8,12,13] is limited by methodological differences. The disparity between these analytical approaches precludes a meaningful correlation, as the latter methods are prone to significant systematic overestimation or underestimation errors that hinder a rigorous comparison of individual (poly)phenol content.

## 4. Conclusions

In summary, this study successfully establishes a robust methodological framework for the design of plant-based beverages with precisely characterized bioactive profiles. By leveraging high-resolution phytochemical mapping through a targeted LC-MS/MS approach with 57 authentic standards, it was demonstrated that ingredient selection can be strategically optimized based on analytical complementarity to maximize (poly)phenolic diversity. The resulting green smoothie—incorporating Granny Smith apple, green celery, green chicory, and peppermint—exhibited a high concentration of six distinct (poly)phenol families, encompassing flavonoids and non-flavonoids, while maintaining excellent sensory attributes. Notably, this high-resolution analysis allowed for the first reporting of seven (poly)phenolic compounds in peppermint leaves, including significant levels of isorhamnetin-3-*O*-rutinoside, which underscores the critical importance of rigorous analytical screening in product formulation.

These findings position fruit and vegetable-based smoothies as effective matrices for enhancing the dietary intake of a broad spectrum of bioactive compounds. However, acknowledging the limited shelf life of unprocessed plant-based matrices, as well as the high sensitivity of (poly)phenols to oxidation, further research is required to evaluate the impact of preservation technologies, such as high pressure and thermal pasteurization [54,55], on the stability and bioavailability of these (poly)phenolic profiles during storage. Ultimately, this comprehensive chemical fingerprinting of smoothie (poly)phenols provides a crucial scientific foundation for accurately designing subsequent studies on bioaccessibility and bioavailability, which are essential to validate the potential health benefits of these precisely formulated food matrices.

## Figures and Tables

**Table 1:** (Poly)phenolic profile of Granny Smith apple, green celery, green chicory, peppermint and the smoothie. Data are expressed as mean ± standard deviation (µg/g dm).

(Poly)Phenolic Compound (µg/g dm)	Granny Smith Apple	Green Celery	Green Chicory	Peppermint	Smoothie
**NON-FLAVONOIDS**					
**Benzoic acids**					
2,5-Dihydroxybenzoic acid	<LoD	<LoD	<LoD	6.71 ± 0.67	<LoD
3,4-Dihydroxybenzoic acid (Protocatechuic acid)	<LoD	<LoD	<LoD	15.51 ± 1.98	0.31 ± 0.03
3,4,5-Trihydroxybenzoic acid (Gallic acid)	<LoD	<LoD	<LoD	1.44 ± 0.07	<LoD
3,5-Dimethoxy-4-hydroxybenzoic acid (Syringic acid)	<LoD	<LoD	<LoD	<LoD	<LoD
Total benzoic acids	<LoD	<LoD	<LoD	23.67 ± 1.21	0.31 ± 0.03
**Hydroxycinnamic acids**					
4′-Hydroxycinnamic acid (*p*-coumaric acid)	<LoD	7.09 ± 0.26	<LoD	6.24 ± 0.05	0.31 ± 0.02
3′,4′-Dihydroxycinnamic acid (Caffeic acid)	<LoD	3.45 ± 0.12	50.65 ± 2.00	82.27 ± 1.25	1.75 ± 0.12
4′-Hydroxy-3′-methoxycinnamic acid (Ferulic acid)	<LoD	3.71 ± 0.29	1.76 ± 0.10	3.57 ± 0.03	0.78 ± 0.04
2-*O*-Caffeoyl-3-(3′,4′-dihydroxyphenyl)lactic acid (Rosmarinic acid)	<LoD	<LoD	<LoD	8196.77 ± 171.50	44.75 ± 1.25
2,3-*O*-Dicaffeoyltartaric acid (Chicoric acid)	<LoD	<LoD	3001.52 ± 214.95	<LoD	152.08 ± 11.12
5-*O*-Caffeoylquinic acid	319.78 ± 7.31	54.60 ± 1.03	1807.62 ± 49.15	22.33 ± 1.54	342.61 ± 9.11
4-*O*-Caffeoylquinic acid	5.44 ± 0.31	0.64 ± 0.04	2.33 ± 0.05	170.01 ± 0.69	4.58 ± 0.09
3,5-*O*-Dicaffeoylquinic acid	<LoD	<LoD	172.92 ± 8.52	16.00 ± 4.17	6.01 ± 0.28
3,4-*O*-Dicaffeoylquinic acid	<LoD	<LoD	<LoD	9.17 ± 4.36	1.32 ± 0.08
4,5-*O*-Dicaffeoylquinic acid	<LoD	<LoD	2.70 ± 0.06	3.99 ± 0.24	<LoD
Total hydroxycinnamic acids	325.23 ± 5.17	69.49 ± 0.50	5039.50 ± 83.41	8510.35 ± 57.21	554.19 ± 4.81
**Phenylethanols**					
2-(3′,4′-Dihydroxyphenyl)ethanol (hydroxytyrosol)	<LoD	<LoD	<LoD	1.50 ± 0.02	<LoD
Total phenylethanols	<LoD	<LoD	<LoD	1.50 ± 0.02	<LoD
**TOTAL NON-FLAVONOIDS**	**325.23 ± 5.17**	**69.49 ± 0.50**	**5039.50 ± 83.41**	**8535.53 ± 47.60**	**554.50 ± 4.56**
**FLAVONOIDS**					
**Flavan-3-ols**					
(−)-Epicatechin	52.03 ± 2.46	<LoD	<LoD	<LoD	88.52 ± 4.62
(+)-Catechin	366.95 ± 7.81	<LoD	<LoD	<LoD	387.00 ± 17.05
Epigallocatechin	<LoD	<LoD	<LoD	<LoD	<LoD
Procyanidin B2 ((−)-epicatechin, (−)-epicatechin)	671.60 ± 3.76	<LoD	<LoD	<LoD	643.78 ± 20.43
Procyanidin B1 ((−)-epicatechin, (+)-catechin)	179.81 ± 2.39	<LoD	<LoD	<LoD	178.81 ± 7.34
Procyanidin C1 ((−)-epicatechin, (−)-epicatechin, (−)-epicatechin)	294.59 ± 23.99	<LoD	<LoD	<LoD	231.58 ± 4.89
Total flavan-3-ols	1564.99 ± 11.51	<LoD	<LoD	<LoD	1529.70 ± 12.71
**Flavonols**					
Kaempferol	<LoD	<LoD	0.38 ± 0.02	<LoD	0.51 ± 0.06
Kaempferol-7-*O*-glucoside	1.17 ± 0.08	<LoD	1.28 ± 0.06	16.52 ± 0.24	1.16 ± 0.10
Kaempferol-3-*O*-glucuronide	<LoD	<LoD	1763.92 ± 97.60	<LoD	85.89 ± 1.98
Kaempferol-3-*O*-rutinoside	<LoD	<LoD	<LoD	<LoD	<LoD
**FLAVONOIDS**					
**Flavonols**					
Quercetin	<LoD	<LoD	<LoD	0.30 ± 0.03	1.19 ± 0.10
Quercetin-3-*O*-arabinoside	7.99 ± 0.89	<LoD	<LoD	<LoD	2.31 ± 0.28
Quercetin-3-*O*-xyloside	57.33 ± 7.80	<LoD	<LoD	<LoD	23.22 ± 0.96
Quercetin-3-*O*-rhamnoside	124.66 ± 17.76	<LoD	<LoD	<LoD	42.08 ± 3.25
Quercetin-3-*O*-glucoside	70.89 ± 7.12	<LoD	<LoD	12.78 ± 1.43	18.38 ± 1.66
Quercetin-3-*O*-galactoside	148.88 ± 21.38	<LoD	<LoD	<LoD	46.15 ± 4.75
Quercetin-3-*O*-rutinoside	27.93 ± 0.51	<LoD	<LoD	7.24 ± 0.50	11.88 ± 1.59
Isorhamnetin	<LoD	<LoD	<LoD	<LoD	<LoD
Isorhamnetin-3-*O*-glucoside	<LoD	<LoD	<LoD	<LoD	<LoD
Isorhamnetin-3-*O*-rutinoside	<LoD	<LoD	<LoD	1007.85 ± 57.09	8.91 ± 0.28
Total flavonols	438.84 ± 11.24	<LoD	1765.58 ± 56.35	1044.69 ± 25.54	241.68 ± 1.99
**Dihydrochalcones**					
Phloretin	<LoD	<LoD	<LoD	<LoD	<LoD
Phloretin-2′-*O*-glucoside	52.20 ± 1.56	<LoD	<LoD	<LoD	24.96 ± 1.19
Total dihydrochalcones	52.20 ± 1.56	<LoD	<LoD	<LoD	24.96 ± 1.19
**Flavones**					
Apigenin	<LoD	<LoD	<LoD	9.99 ± 0.98	0.66 ± 0.07
Apigenin-7-*O*-glucoside	<LoD	0.42 ± 0.02	<LoD	3.77 ± 0.11	<LoD
Apigenin-7-*O*-glucuronide	<LoD	<LoD	<LoD	81.50 ± 2.59	0.69 ± 0.08
Apigenin-7-*O*-rutinoside	<LoD	<LoD	<LoD	1040.09 ± 54.64	10.77 ± 0.28
Apigenin-7-(2-*O*-apiosylglucoside)	<LoD	12.88 ± 0.48	<LoD	<LoD	0.58 ± 0.02
Apigenin-8-*C*-glucoside	<LoD	<LoD	<LoD	<LoD	<LoD
Apigenin-6,8-*C*-diglucoside	<LoD	<LoD	<LoD	28.68 ± 2.58	4.55 ± 0.11
Luteolin	<LoD	<LoD	<LoD	96.63 ± 3.68	12.16 ± 0.46
Luteolin-7-*O*-glucoside	<LoD	0.36 ± 0.01	<LoD	43.54 ± 1.70	0.82 ± 0.05
Luteolin-8-*C*-glucoside	<LoD	<LoD	<LoD	1.91 ± 0.32	<LoD
Luteolin-7-*O*-glucuronide	<LoD	<LoD	<LoD	3994.71 ± 455.17	30.69 ± 0.97
Diosmetin	<LoD	<LoD	<LoD	15.62 ± 1.38	0.75 ± 0.02
Diosmetin-7-*O*-glucoside	<LoD	<LoD	<LoD	8.52 ± 0.45	<LoD
Diosmetin-7-*O*-rutinoside	<LoD	<LoD	<LoD	2367.85 ± 272.46	33.61 ± 2.85
Total flavones	<LoD	13.65 ± 0.28	<LoD	7692.82 ± 153.96	95.29 ± 0.97
**Flavanones**					
Naringenin-7-*O*-rutinoside	<LoD	<LoD	<LoD	353.04 ± 4.96	4.75 ± 0.16
Isosakuranetin-7-*O*-rutinoside	<LoD	<LoD	<LoD	110.25 ± 11.53	1.06 ± 0.08
Eriodictyol	<LoD	<LoD	<LoD	394.26 ± 18.42	11.20 ± 0.48
Eriodictyol-7-*O*-rutinoside	<LoD	<LoD	<LoD	43,473.70 ± 5436.81	380.97 ± 7.72
Hesperetin	<LoD	<LoD	<LoD	37.06 ± 0.69	1.61 ± 0.20
Hesperetin-7-*O*-rutinoside	<LoD	<LoD	<LoD	4909.66 ± 733.73	101.97 ± 4.23
Total flavanones	<LoD	<LoD	<LoD	49,277.98 ± 2239.71	501.55 ± 3.60
**TOTAL FLAVONOIDS**	**2056.02 ± 10.92**	**13.65 ± 0.28**	**1765.58 ± 56.35**	**58 , 015.49 ± 1149.39**	**2393.18 ± 5.33**
**TOTAL PHENOLIC COMPOUNDS**	**2381.25 ± 10.34**	**83.15 ± 0.43**	**6805.08 ± 76.30**	**66,551.02 ± 919.16**	**2947.68 ± 5.17**

<LoD; below the limit of detection. The LoD for each compound is provided in Table S1.

**Table 2:** Composition of the smoothies formulated based on descriptive analysis (expressed as g/100 g smoothie).

(g/100 g Smoothie)	Granny Smith Apple Juice	Granny Smith Apple	Green Celery	Dried Green Chicory	Dried Peppermint Leaves
Formulation 1	32.0	32.0	32.4	0.8	0.1
Formulation 2	36.2	30.0	30.0	1.0	0.1
Formulation 3	36.7	30.0	30.0	0.5	0.1
Formulation 4	30.0	41.2	25.0	1.0	0.1
Formulation 5	30.0	41.7	25.0	0.5	0.1

## Data Availability

The original contributions presented in this study are included in the article and Supplementary Materials. Further inquiries can be directed to the corresponding author.

## Supplementary Materials

The following supporting information can be downloaded at https://www.mdpi.com/article/10.3390/foods15081293/s1, Table S1: Mass spectrometric identification and quantification parameters of (poly)phenolic compounds analyzed by LC-MS/MS, their recommended nomenclature proposed by Kay et al. [17] and other common names; Table S2: Sensory sheet used in the descriptive analysis carried out during the smoothie formulation development; Table S3: (Poly)phenolic profile of Swiss chard and borage; Figure S1: Mass chromatogram (MRM) of pure standard mix at 2.5 µg/mL; Figure S2: Mass chromatogram (MRM) of Swiss chard; Figure S3: Mass chromatogram (MRM) of borage; Figure S4: Mass chromatogram (MRM) of Granny Smith apple; Figure S5: Mass chromatogram (MRM) of green celery; Figure S6: Mass chromatogram (MRM) of green chicory; Figure S7: Mass chromatogram (MRM) of peppermint; Figure S8: Mass chromatogram (MRM) of smoothie formulation.

## Abbreviations

The following abbreviations are used in this manuscript:

HPLC	High-performance liquid chromatography
LC	Liquid chromatography
MS/MS	Tandem mass spectrometry
