# Supplementary material for: Design of a Plant-Based Smoothie: Exploiting Ingredient Complementarity for a Diversified (Poly)phenolic Profile Quantified by Targeted LC-MS/MS Analysis
Source: Foods. 2026 Apr 9;15(8):1293. doi: 10.3390/foods15081293 (PMC13114553; doi:10.3390/foods15081293)
Supplement: Supplementary file 1 [file foods-15-01293-s001.zip › foods-4181033-supplementary.pdf]

TITLE: Design of a Plant-Based Smoothie: Exploiting Ingredient Complementarity for a Diversified (Poly)phenolic Profile Quantified by Targeted LC-MS/MS Analysis

Cristina Matías [1,2], Cristina Del Burgo-Gutiérrez [1,3], María-José Sáiz-Abajo [2], María-Paz De Peña [1,3,4], Iziar A. Ludwig [1,3,4,*] and Concepción Cid [1,3,4]

[1] University of Navarra, Faculty of Pharmacy and Nutrition, Centre for Nutrition Research and Department of Nutrition, Food Science and Physiology, C/Irunlarrea 1, 31008 Pamplona, Navarra, Spain; cdelb.7@unav.es (C.D.B.-G.), mpdepena@unav.es (M.-P.P.), iludwig@unav.es (I.A.L.), ccid@unav.es (C.C.)

[2] National Centre for Food Technology and Safety (CNTA). NA 134, Km. 53. 31570 San Adrián, Navarra, Spain. cmatias@cnta.es (C.M.), mjsaiz@cnta.es (M.-J.S.-A.)

[3] Universidad de Navarra, Instituto de Nutrición y Salud (INS), Campus Universitario, 31009, Pamplona, España.

[4] IdiSNA, Navarra Institute for Health Research, Pamplona, Spain.

* Correspondence: iludwig@unav.es; Tel.: +34 948 425600 (Ext. 806652)

SUPPLEMENTARY MATERIAL

Table S1. Mass spectrometric identification and quantification parameters of (poly)phenolic compounds analyzed by LC-MS/MS, their recommended nomenclature proposed by Kay et al. [17] and other common names.

Recommended Name	Other Common Names	Rt (min)	$[M-H]^-$ (m/z)	MS/MS Fragmentation	CE (eV)	LoD (µg/g)	LoQ (µg/g)	Calibration Range (µg/mL)
**NON-FLAVONOIDS**								
**Benzoic acids**								
2,5-Dihydroxybenzoic acid	-	4.4	153	108, 109	-30	0.23	0.75	0.025-1.0
3,4-Dihydroxybenzoic acid	Protocatechuic acid	2.2	153	109, 91, 81	-35	0.09	0.30	0.025-0.75
3,4,5-Trihydroxybenzoic acid	Gallic acid	0.9	169	125	-20	0.09	0.30	0.01-0.75
3,5-Dimethoxy-4-hydroxybenzoic acid	Syringic acid	6.5	197	153, 182	-16	0.45	1.50	0.05-2.5
**Hydroxycinnamic acids**								
4´-Hydroxycinnamic acid	*p*-coumaric	8.7	163	119, 93	-30	0.09	0.30	0.01-0.50
3´,4´-Dihydroxycinnamic acid	Caffeic acid	6.1	179	135, 134	-30	0.02	0.08	0.05-0.75
4´-Hydroxy-3´-methoxycinnamic acid	Ferulic acid	9.5	193	134, 178	-20	0.23	0.75	0.05-0.75
2-*O*-Caffeoyl-3-(3´,4´-dihydroxyphenyl)lactic acid	Rosmarinic acid	13.3	359	161, 197	-22	0.23	0.75	0.025-1.0
2,3-*O*-Dicaffeoyltartaric acid	Chicoric acid	9.9	473	149, 135	-26	0.90	3.00	0.10-1.0
5-*O*-Caffeoylquinic acid	Chlorogenic acid	5.5	353	191, 179	-20	0.02	0.08	0.05-2.5
4-*O*-Caffeoylquinic acid	Cryptochlorogenic acid	6.4	353	173, 179	-30	0.09	0.30	0.01-2.5
3,5-*O*-Dicaffeoylquinic acid	-	12.1	515	353, 191	-30	0.23	0.75	0.025-1.0
3,4-*O*-Dicaffeoylquinic acid	-	11.6	515	353, 335, 299	-30	0.02	0.08	0.01-1.0
4,5-*O*-Dicaffeoylquinic acid	-	13.2	515	353, 173	-30	0.09	0.30	0.01-1.0
**Phenylethanols**								
2-(3´,4´-Dihydroxyphenyl)ethanol	Hydroxytyrosol	2.1	153	123, 108	-30	0.02	0.08	0.01-0.75

Recommended name	Other common names	Rt (min)	$[M-H]^-$ (m/z)	MS/MS fragmentation	CE (eV)	LoD (µg/g)	LoQ (µg/g)	Calibration range (µg/mL)
**FLAVONOIDS**								

**Flavan-3-ols**								
(-)-Epicatechin	-	5.7	289	245, 109	-18	0.09	0.30	0.01-2.5
(+)-Catechin	-	8.2	289	245, 109	-18	0.09	0.30	0.01-2.5
Epigallocatechin	-	5.1	30	125, 179	-28	2.25	7.50	0.25-2.5
Procyanidin B_2 ((-)-epicatechin, (-)-epicatechin)	-	7.5	577	289, 125	-32	0.09	0.30	0.01-2.5
Procyanidin B_1 ((-)-epicatechin, (+)-catechin)	-	4.9	577	289, 125	-32	0.09	0.30	0.01-1.0
Procyanidin C_1 ((-)-epicatechin, (-)-epicatechin, (-)-epicatechin)	-	8.9	865	125, 287	-76	0.09	0.30	0.01-2.5
**Flavonols**								
Kaempferol	-	18.1	285	93, 65	-50	0.02	0.08	0.05-2.5
Kaempferol-7-*O*-glucoside	-	12.3	447	285, 284, 151	-30	0.09	0.30	0.01-0.50
Kaempferol-3-*O*-glucuronide	-	12.0	461	285, 113	-28	0.23	0.75	0.025-2.5
Kaempferol-3-*O*-rutinoside	-	11.0	593	285, 255, 227	-50	0.09	0.30	0.01-0.75
Quercetin	-	15.7	301	151, 179	-30	0.09	0.30	0.025-0.50
Quercetin-3-*O*-arabinoside	Guaiaverin	10.9	433	300, 271	-32	0.09	0.30	0.01-2.5
Quercetin-3-*O*-xyloside	Reynoutrin	10.5	433	300, 301	-30	0.45	1.50	0.05-2.5
Quercetin-3-*O*-rhamnoside	Quercitrin	12.0	447	301, 283	-40	0.23	0.75	0.025-2.5
Quercetin-3-*O*-glucoside	Isoquercitrin	10.1	463	300, 301, 271	-35	0.23	0.75	0.025-1.0
Quercetin-3-*O*-galactoside	Hyperoside	9.9	463	300, 301	-36	0.45	1.50	0.05-2.5
Quercetin-3-*O*-rutinoside	Rutin	9.8	609	301, 179	-50	0.68	2.25	0.075-1.0
Isorhamnetin	-	18.2	315	300, 151	-30	0.09	0.30	0.05-0.75

Recommended name	Other common names	Rt (min)	$[M-H]^-$ (m/z)	MS/MS fragmentation	CE (eV)	LoD (µg/g)	LoQ (µg/g)	Calibration range (µg/mL)
**Flavonols**								
Isorhamnetin-3-*O*-glucoside	-	12.2	477	314, 271, 285	-25	0.09	0.30	0.01-0.5
Isorhamnetin-3-*O*-rutinoside	-	11.5	623	577, 315	-30	0.45	1.50	0.05-1.0

Recommended name	Other common names	Rt (min)	$[M-H]^-$ (m/z)	MS/MS fragmentation	CE (eV)	LoD (µg/g)	LoQ (µg/g)	Calibration range (µg/mL)
**Dihydrochalcones**								
Phloretin	-	17.9	273	167, 123	-22	0.09	0.30	0.01-0.75
Phloretin-2´-*O*-glucoside	Phloridzin	13.6	435	273, 167	-22	0.45	1.50	0.05-2.5
**Flavones**								
Apigenin	-	17.6	269	151, 149, 117	-30	0.02	0.08	0.05-0.75
Apigenin-7-*O*-glucoside	-	12.3	431	269, 311, 283	-40	0.09	0.30	0.025-0.75
Apigenin-7-*O*-glucuronide	-	12.7	445	269, 113, 85	-30	0.09	0.30	0.025-0.75
Apigenin-7-*O*-rutinoside	Isorhoifolin	11.5	577	269	-40	0.09	0.30	0.01-0.75
Apigenin-7-(2-*O*-apiosylglucoside)	Apiin	12.0	563	269, 118	-50	0.09	0.30	0.075-2.5
Apigenin-8-*C*-glucoside	Vitexin	9.8	431	311, 283	-30	0.09	0.30	0.05-0.75
Apigenin-6,8-*C*-diglucoside	Vicenin-2	8.4	593	353, 383, 473	-45	0.23	0.75	0.025-1.0
Luteolin	-	15.6	285	133, 151	-50	0.02	0.08	0.075-0.75
Luteolin-7-*O*-glucoside	Cynaroside	10.2	447	285, 327	-30	0.09	0.30	0.05-0.5
Luteolin-8-*C*-glucoside	Orientin	9.4	447	327, 297, 285	-35	0.09	0.30	0.01-0.75
Luteolin-7-*O*-glucuronide	-	10.2	461	285, 327	-30	0.45	1.50	0.05-1.0
Diosmetin	-	18.1	299	284, 256	-40	0.09	0.30	0.05-0.75
Diosmetin-7-*O*-glucoside	-	13.1	461	299, 284	-35	0.09	0.30	0.01-0.75
Diosmetin-7-*O*-rutinoside	Diosmin	12.4	607	299, 284	-34	0.23	0.75	0.025-2.5
**Flavanones**								
Naringenin-7-*O*-rutinoside	Narirutin	11.1	579	271, 151	-30	0.02	0.08	0.01-1.0
Isosakuranetin-7-*O*-rutinoside	Didymin	15.6	593	285, 309	-30	0.23	0.75	0.025-0.75
Eriodictyol	-	15.3	287	151, 135	-20	0.45	1.50	0.05-1.0
Eriodictyol-7-*O*-rutinoside	Eriocitrin	9.8	595	287, 151	-32	2.25	7.50	0.25-2.5
Hesperetin	-	18.1	301	164, 108	-32	0.45	1.50	0.075-1.0
Hesperetin-7-*O*-rutinoside	Hesperedin	12.5	609	301, 342	-30	0.23	0.75	0.025-1.0

Rt, retention time; m/z, mass-to-charge ratio; $[M–H]^–$, Negatively charged molecular ion; CE, Collision energy.

Table S2. Sensory sheet used in the descriptive analysis carried out during the smoothie formulations development.

**Appearance**
Color intensity*
*Color scale: 1.- Yellow, 2.- Yellow- greenish, 3.- Green, 4.- Dark green, 5.- Brownish-green, 6.- Brown
Defect (suspended solids, phase separation…)
**Odor**
Odor intensity
Off-odor
**Taste/Flavor**
Taste intensity
Sweetness
Acidity
Bitterness
Persistence in mouth
Astringency
Off-taste/off-flavor
**Texture**
Consistency
Presence of lumps and/or fibers, sandiness, etc.

Scale: 1.- Absence, 2.- Light, 3.- Moderate, 4.- Adequate presence, 5.- Intense, 6.- Very intense, 7.- Extremely intense

Table S3. (Poly)phenolic profile of Swiss chard and borage.

	**Swiss chard**	**Borage**
**NON-FLAVONOIDS** (μg/g dm)		
**Benzoic acids**		
2,5-Dihydroxybenzoic acid	<LoD	<LoD
3,4-Dihydroxybenzoic acid (Protocatechuic acid)	<LoD	0.33 ± 0.02
3,4,5-Trihydroxybenzoic acid (Gallic acid)	<LoD	<LoD
3,5-Dimethoxy-4-hydroxybenzoic acid (Syringic acid)	<LoD	<LoD
**Hydroxycinnamic acids**		
4´-Hydroxycinnamic acid (*p*-coumaric acid)	3.05 ± 0.17	3.49 ± 0.27
3´,4´-Dihydroxycinnamic acid (Caffeic acid)	0.24 ±0.01	28.79 ± 1.56
4´-Hydroxy-3´-methoxycinnamic acid (Ferulic acid)	6.72 ± 0.58	0.79 ± 0.05
2-*O*-Caffeoyl-3-(3´,4´-dihydroxyphenyl)lactic acid (Rosmarinic acid)	<LoD	<LoD
2,3-*O*-Dicaffeoyltartaric acid (Chicoric acid)	<LoD	<LoD
5-*O*-Caffeoylquinic acid	0.16 ± 0.01	<LoD
4-*O*-Caffeoylquinic acid	<LoD	<LoD
3,5-*O*-Dicaffeoylquinic acid	<LoD	<LoD
3,4-*O*-Dicaffeoylquinic acid	0.22 ± 0.02	<LoD
4,5-*O*-Dicaffeoylquinic acid	0.65 ± 0.04	<LoD
**Phenylethanols**		
2-(3´,4´-Dihydroxyphenyl)ethanol (Hydroxytyrosol)	<LoD	0.09 ± 0.00
**TOTAL NON-FLAVONOIDS**	11.04 ± 0.98	33.49 ± 2.06
**FLAVONOIDS** (μg/g sample dm)		
**Flavan-3-ols**		
(-)-Epicatechin	<LoD	<LoD
(+)-Catechin	<LoD	<LoD
Epigallocatechin	<LoD	<LoD
Procyanidin B2 ((-)-epicatechin, (-)-epicatechin)	<LoD	<LoD
Procyanidin B1 ((-)-epicatechin, (+)-catechin)	<LoD	<LoD
Procyanidin C1 ((-)-epicatechin, (-)-epicatechin, (-)-epicatechin)	<LoD	<LoD
**Flavonols**		
Kaempferol	0.48 ± 0.02	0.15 ± 0.01
Kaempferol-7-*O*-glucoside	<LoD	<LoD
Kaempferol-3-*O*-glucuronide	<LoD	<LoD
Kaempferol-3-*O*-rutinoside	0.42 ± 0.03	1.81 ± 0.14
Quercetin	0.43 ± 0.02	<LoD
Quercetin-3-*O*-arabinoside	<LoD	<LoD
Quercetin-3-*O*-xyloside	<LoD	<LoD
Quercetin-3-*O*-rhamnoside	<LoD	<LoD
Quercetin-3-*O*-glucoside	2.5 ± 0.09	<LoD
Quercetin-3-*O*-galactoside	<LoD	<LoD
Quercetin-3-*O*-rutinoside	6.89 ± 0.24	<LoD
**FLAVONOIDS** (μg/g sample dm)	**Swiss chard**	**Borage**
**Flavonols**		
Isorhamnetin	1.87 ± 0.11	0.42 ± 0.02

Isorhamnetin-3-*O*-glucoside	6.28 ± 0.37	<LoD
Isorhamnetin-3-*O*-rutinoside	<LoD	<LoD
**Dihydrochalcones**		
Phloretin	<LoD	<LoD
Phloretin-2´-*O*-glucoside	<LoD	<LoD
**Flavones**		
Apigenin	0.12 ± 0.01	0.08 ± 0.00
Apigenin-7-*O*-glucoside	<LoD	<LoD
Apigenin-7-*O*-glucuronide	<LoD	<LoD
Apigenin-7-*O*-rutinoside	<LoD	<LoD
Apigenin-7-(2-*O*-apiosylglucoside)	<LoD	<LoD
Apigenin-8-*C*-glucoside	1.05 ± 0.06	<LoD
Apigenin-6,8-*C*-diglucoside	<LoD	<LoD
Luteolin	0.15 ± 0.01	<LoD
Luteolin-7-*O*-glucoside	<LoD	<LoD
Luteolin 8-*C*-glucoside	<LoD	<LoD
Luteolin-7-*O*-glucuronide	<LoD	<LoD
Diosmetin	3.32 ± 0.23	0.31 ± 0.02
Diosmetin-7-*O*-glucoside	9.01 ± 0.57	<LoD
Diosmetin-7-*O*-rutinoside	<LoD	<LoD
**Flavanones**		
Naringenin-7-*O*-glucoside	0.08 ± 0.01	<LoD
Isosakuranetin-7-*O*-rutinoside	<LoD	<LoD
Eriodictyol	<LoD	<LoD
Eriodictyol-7-*O*-rutinoside	<LoD	<LoD
Hesperetin	<LoD	<LoD
Hesperetin-7-*O*-rutinoside	13.69 ± 0.98	4.76 0.34
**TOTAL FLAVONOIDS**	46.29 ± 2.54	7.53 ± 0.46
**TOTAL PHENOLIC COMPOUNDS**	57.33 ± 2.98	41.02 ±2.99

<LoD; below the limit of detection. The LoD for each compound is provided in Table S1.

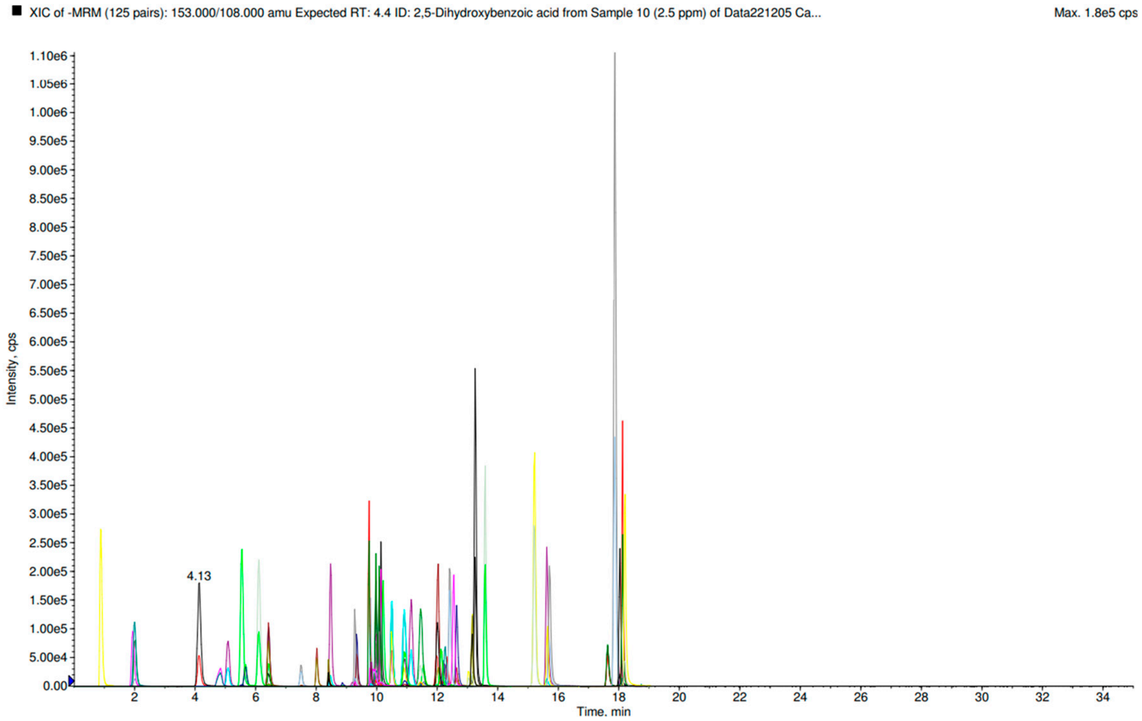

Figure S1. Mass chromatogram (MRM) of pure standard mix at 2,5 µg/mL

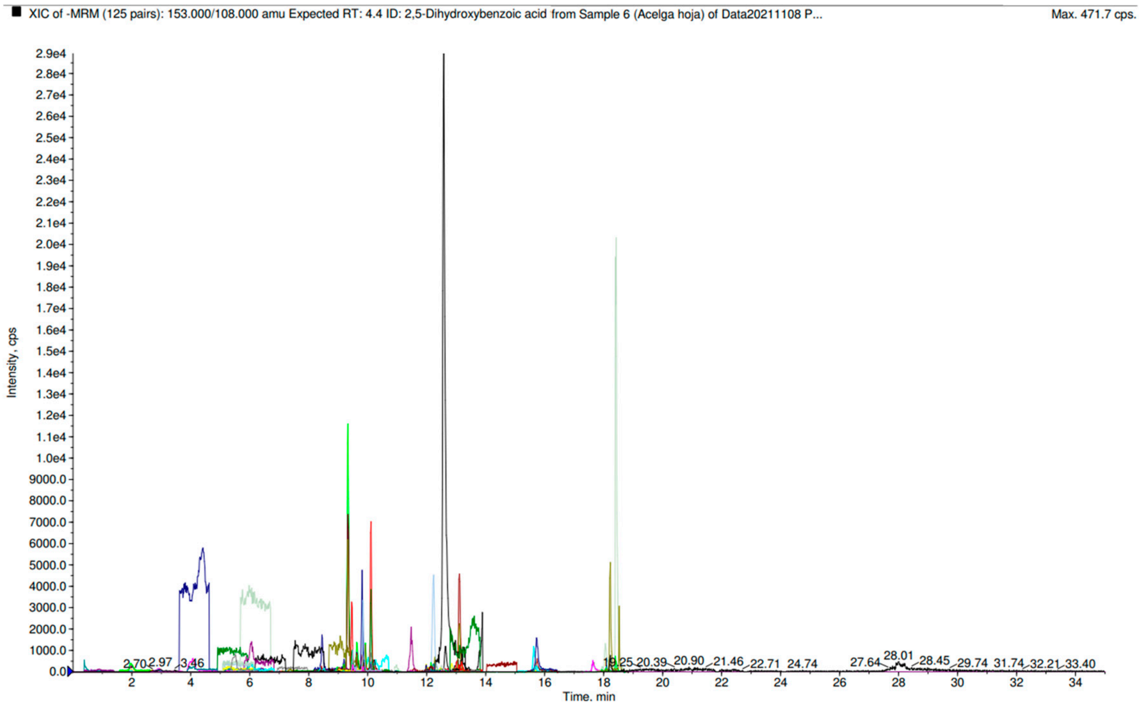

Figure S2. Mass chromatogram (MRM) of Swiss chard.

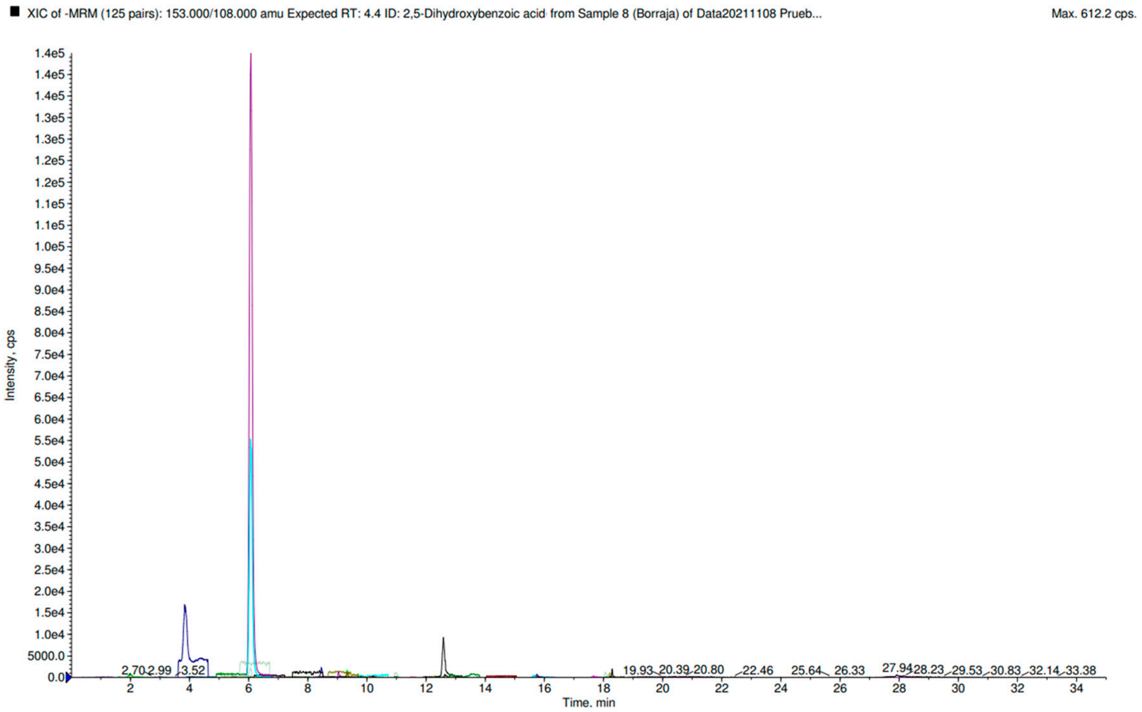

Figure S3. Mass chromatogram (MRM) of borage.

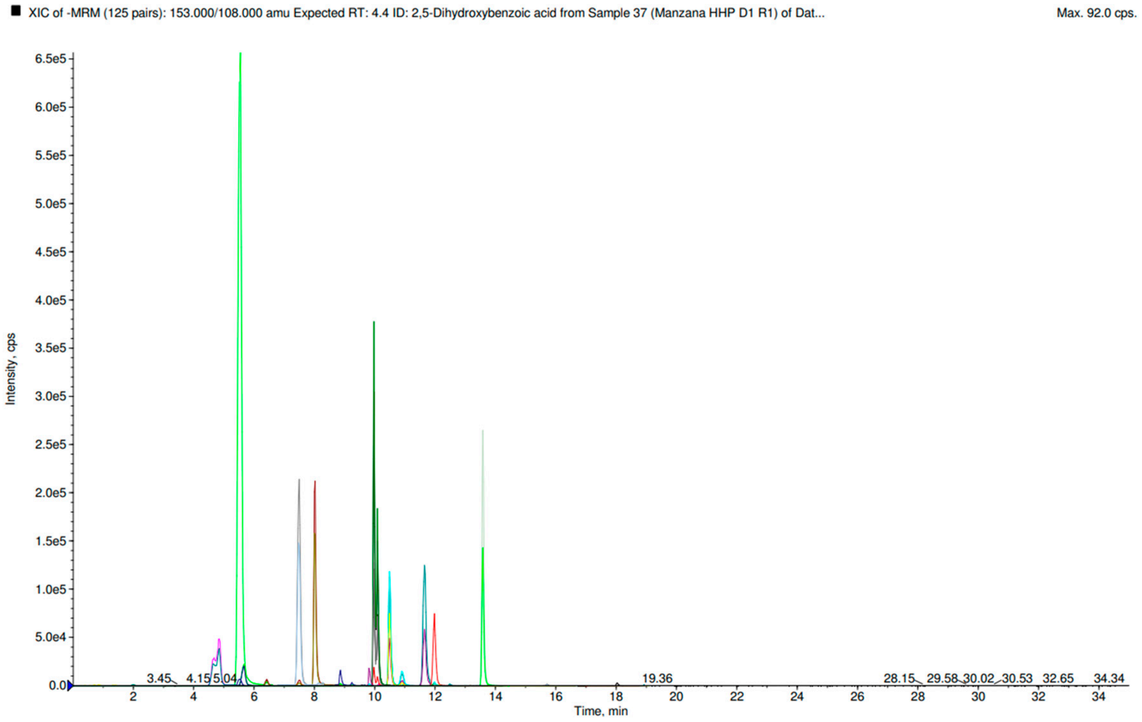

Figure S4. Mass chromatogram (MRM) of Granny Smith apple.

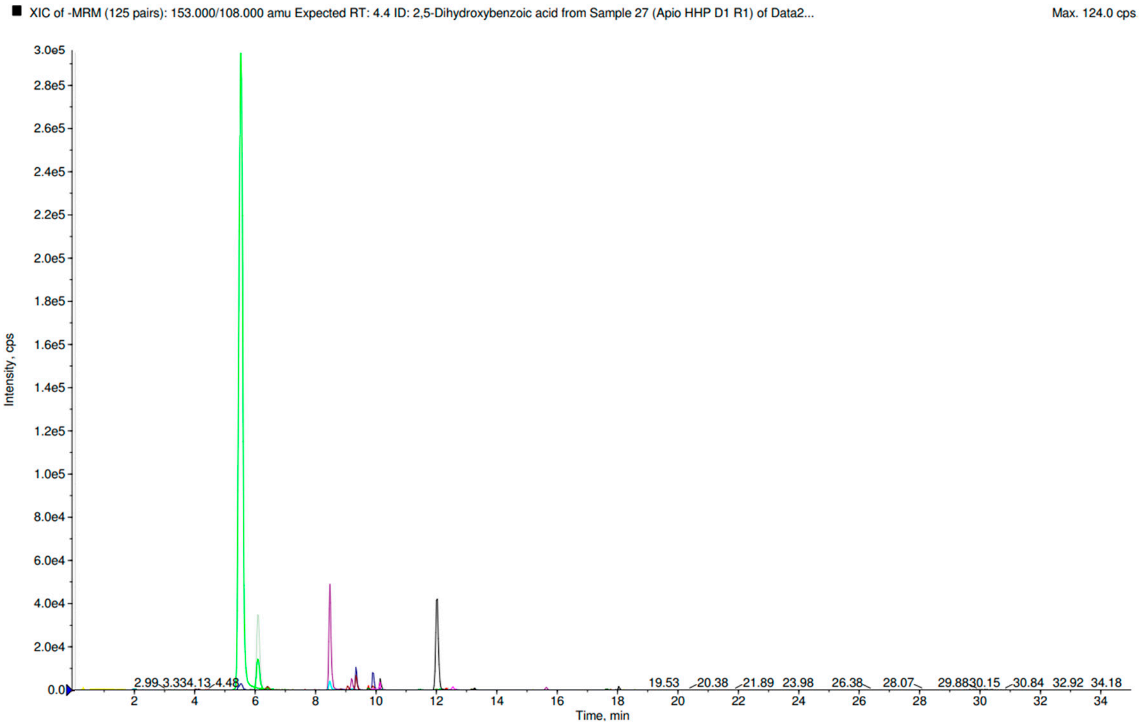

Figure S5. Mass chromatogram (MRM) of green celery.

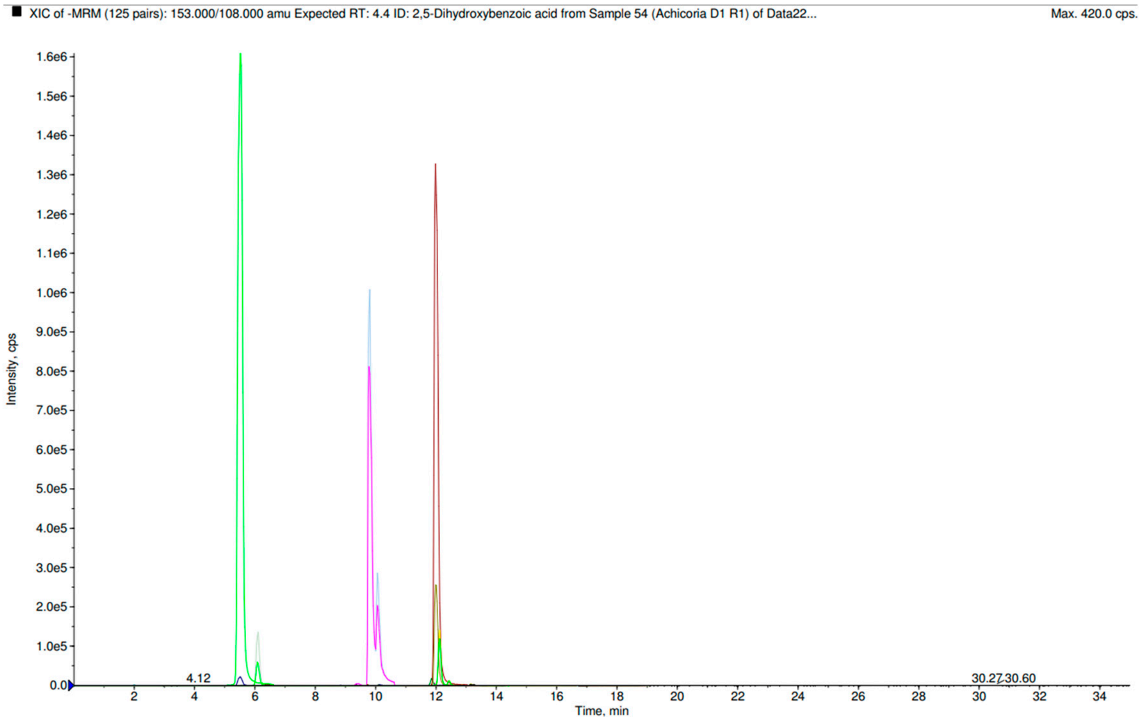

Figure S6. Mass chromatogram (MRM) of green chicory.

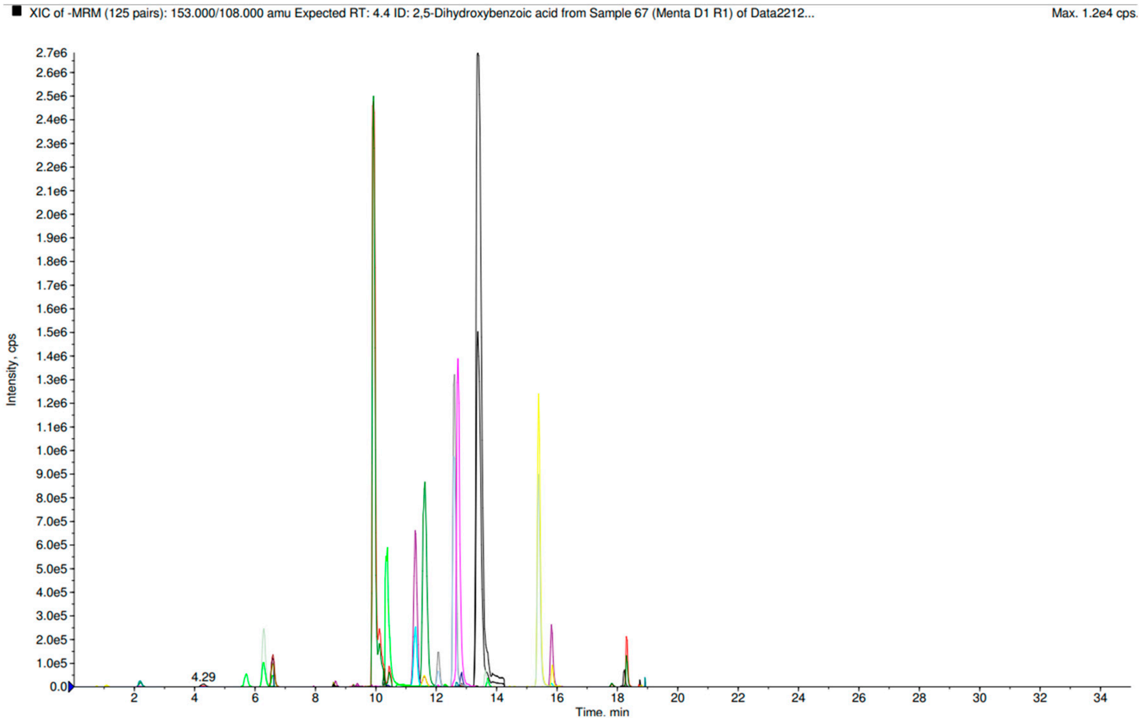

Figure S7. Mass chromatogram (MRM) of peppermint.

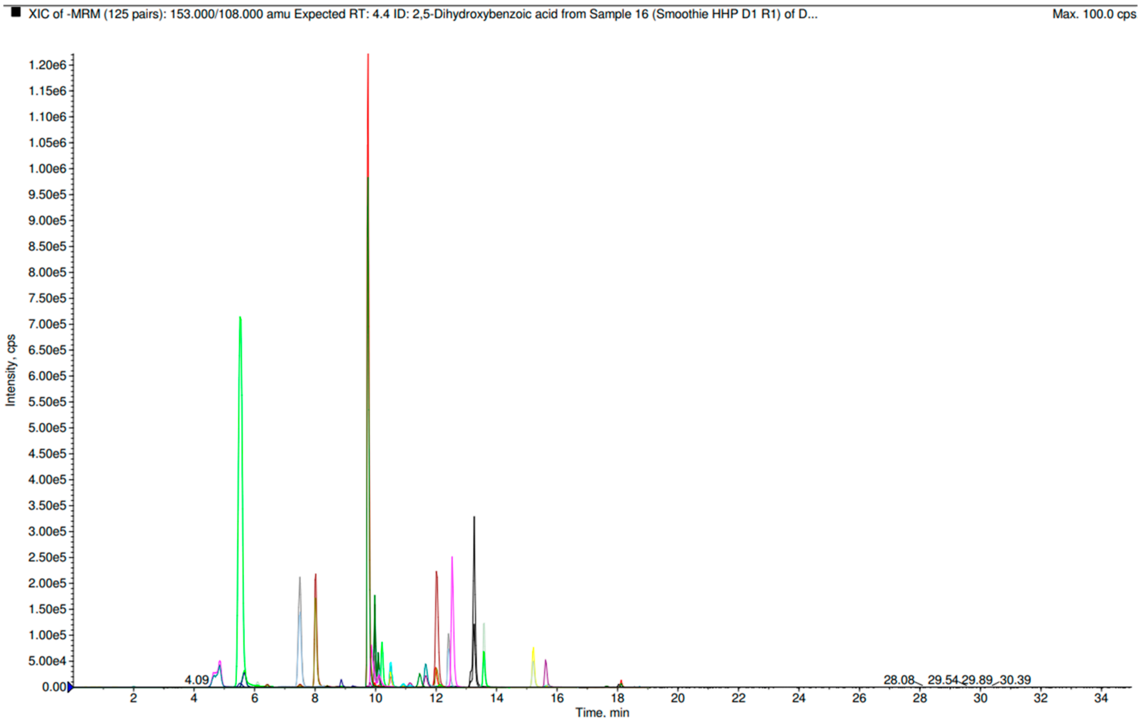

Figure S8. Mass chromatogram (MRM) of smoothie formulation.
